# Feasibility of a Multimodal Telemedical Intervention for Patients with Parkinson’s Disease—A Pilot Study

**DOI:** 10.3390/jcm11041074

**Published:** 2022-02-18

**Authors:** Jonas Bendig, Anna-Sophie Wolf, Tony Mark, Anika Frank, Josephine Mathiebe, Madlen Scheibe, Gabriele Müller, Marcus Stahr, Jochen Schmitt, Heinz Reichmann, Kai F. Loewenbrück, Björn H. Falkenburger

**Affiliations:** 1 Department of Neurology, University Hospital Carl Gustav Carus and Carl Gustav Carus Faculty of Medicine, Technische Universität Dresden, 01307 Dresden, Germany; jonas.bendig@ukdd.de (J.B.); anna-sophie.wolf@ukdd.de (A.-S.W.); tony.mark@ukdd.de (T.M.); anika.frank@ukdd.de (A.F.); marcus.stahr@freenet.de (M.S.); heinz.reichmann@ukdd.de (H.R.); kai.loewenbrueck@ukdd.de (K.F.L.); 2 German Center for Neurodegenerative Diseases (DZNE), 01307 Dresden, Germany; 3 Center for Evidence-Based Healthcare, University Hospital Carl Gustav Carus and Carl Gustav Carus Faculty of Medicine, Technische Universität Dresden, 01307 Dresden, Germany; josephine.mathiebe@ukdd.de (J.M.); madlen.scheibe@ukdd.de (M.S.); gabriele.mueller@ukdd.de (G.M.); jochen.schmitt@ukdd.de (J.S.); 4 Department of Psychiatry, Sächsisches Krankenhaus Arnsdorf, 01477 Arnsdorf, Germany

**Keywords:** telemedicine, telemonitoring, Parkinson disease, usability, user-centered design, sensors, camera, smartphone, mobile applications

## Abstract

Symptoms of Parkinson’s disease (PD) can be controlled well, but treatment often requires expert judgment. Telemedicine and sensor-based assessments can allow physicians to better observe the evolvement of symptoms over time, in particular with motor fluctuations. In addition, they potentially allow less frequent visits to the expert’s office and facilitate care in rural areas. A variety of systems with different strengths and shortcomings has been investigated in recent years. We designed a multimodal telehealth intervention (TelePark) to mitigate the shortcomings of individual systems and assessed the feasibility of our approach in 12 patients with PD over 12 weeks in preparation for a larger randomized controlled trial. TelePark uses video visits, a smartphone app, a camera system, and wearable sensors. Structured training included setting up the equipment in patients’ homes and group-based online training. Usability was assessed by questionnaires and semi-standardized telephone interviews. Overall, 11 out of 12 patients completed the trial (5 female, 6 male). Mean age was 65 years, mean disease duration 7 years, mean MoCA score 27. Adherence was stable throughout the study and 79% for a short questionnaire administered every second day, 62% for medication confirmation, and 33% for an electronic Hauser diary. Quality of life did not change in the course of the study, and a larger cohort will be required to determine the effect on motor symptoms. Interviews with trial participants identified motivations to use such systems and areas for improvements. These insights can be helpful in designing similar trials.

## 1. Introduction

Parkinson’s disease (PD) is the second most common neurodegenerative disease. Between 1990 and 2016, the global prevalence more than doubled, and current models from Western industrialized nations continue to predict rapidly increasing patient numbers in the coming decades [1,2,3]. Especially in rural areas, patients often lack access to a movement disorder specialist or have to travel over long distances to find adequate care [4]. Even in the US and Europe, more than 40% of patients with PD do not consult a PD specialist or neurologist, which results in a higher risk of disease-related complications and mortality [5]. These shortcomings in the current management of patients with PD in combination with the predicted increase in patient numbers require novel approaches [5]. In this context, digital solutions have the potential to improve patient care without binding more expert time. A variety of different systems and approaches has been investigated in recent years. The majority of studies used wearable sensors [6,7,8] or smartphone apps [9,10] to monitor motor symptoms; video-based approaches were used mainly for virtual consultations [11,12]. Recent studies have also investigated automated video analyses [13,14,15]. Each of these solutions has different strengths and shortcomings: Wearable sensors allow for long-term measurements of fluctuating motor symptoms, but can be stigmatizing and burdensome in daily life [16]. Video-based approaches are more comparable to current clinical practice, but are limited to snapshots in time and exclude the evaluation of rigidity as well as postural stability [17]. Smartphone apps can potentially capture many PD symptoms through a combination of passive sensing, active tests, or digital symptom diaries, but can be hard to use for elderly patients and are susceptible to confounders like individual smartphone use [18]. The use of telehealth tools also has the potential to improve clinical decisions [6,19,20]. However, it remains unclear how different components integrate into patients’ everyday lives, how adherence can be optimized, and what kind of additional burden is still feasible. Moreover, there is a lack of randomized controlled trials (RCTs) investigating the effect of telehealth interventions on relevant clinical outcomes like quality of life in patients with PD [21]. We, therefore, designed a multimodal digital intervention with video visits, a camera system, wearable sensors, and a smartphone app to mitigate the above-mentioned drawbacks. Before conducting the RCT, we performed a pilot phase to test the feasibility and patient satisfaction of our approach in a real-life environment and identify potential barriers. The results of the pilot phase are reported here.

## 2. Materials and Methods

### 2.1. Aim of the Study

The aim of this study was to determine whether a multimodal telemedical intervention is feasible in patients with PD and to identify barriers for handling and usability of its components. Feasibility was operationalized as the adherence of participants to the intended use of the components according to the study protocol. We used 68% as the target for completion rates based on the median adherence in a comparable study with smartphones and wearable sensors [22]. Potential barriers were identified in two telephone interviews. We anticipated attrition to be a major barrier to long-term use, and therefore evaluated completion rates and support contacts over time; in addition, we asked patients for overall satisfaction, effort, and integration into their daily life (see below for details). In order to determine whether the intervention can have any efficacy, we recorded the number of therapeutic decisions during the study period. Changes in the health-related quality of life were assessed as an exploratory endpoint using the Parkinson’s Disease Questionnaire (PDQ-39) [23,24] at the beginning and the end of the study.

### 2.2. Participants

Twelve patients were recruited at the University Hospital Carl Gustav Carus Dresden in February and March 2021. The study was approved by the institutional review board of Technische Universität Dresden, Germany (BO-EK-321072020 and BO-EK-114022021). Written informed consent was obtained from all participants and relatives before inclusion in the study. Inclusion criteria were the clinically probable diagnosis of idiopathic Parkinson’s disease by a specialist in movement disorders according to the diagnostic criteria of the International Movement Disorders Society [25] as well as sufficient German language skills. Exclusion criteria were advanced dementia (defined by a Montreal Cognitive Assessment score <21, [26]) and cognitive inability to use the study equipment. Patients were not randomized, and each participant performed the study procedures stated below.

### 2.3. Description of the Telemedical Intervention

All telemedical components were provided free of charge and returned at the end of the study. Patients received an Android smartphone (Nokia 5.3, Nokia Corporation, Espoo, Finnland) equipped with a SIM card for free internet and telephone use with the TelePark-App preinstalled. All partners involved in the study are subject to the European General Data Protection Regulation.

#### 2.3.1. TelePark-App and Patient Management Platform

A smartphone app (TelePark-App; intecsoft group, Dresden, Germany) and a web-based patient management platform (Institut für angewandte Informatik e. V., Leipzig, Germany) were the two key elements of the study intervention, and both were specifically developed for this purpose. The TelePark-App included (i) a chat function, (ii) a medication reminder and medication affirmation, (iii) a digitized Hauser-Diary (motor status every 30 min for three days in a row), (iv) a self-developed short questionnaire to document falls, pain, mood and motor status of the day and (v) a self-developed short touchscreen-based test battery for motor and cognitive function: finger tapping, spiral-drawing, trail making-test, stroop-test, and go/no-go-test. The acquired data was transferred to the patient management platform. The platform included an individual medication plan and task management, which could be pushed to the patient’s phone (for tasks in the TelePark-App, camera-system, and wearable sensor measurements), as well as a chat function.

#### 2.3.2. Video Visits

The video consultation tool was originally planned to be integrated as a part of the TelePark-App, but could not be realized due to technical difficulties. Therefore, video visits used an external software that was developed for medical-purpose video telephony (RED connect Videosprechstunde; RED Medical Systems GmbH). Patients received two appointments for a scheduled video visit during the study which included a structured neurological history as well as an MDS-UPDRS III assessment, omitting items for rigidity and postural stability. During the video visits, the treating physician (JB) made treatment choices based on the reports from the wearable sensors (see Section 2.3.3), videos recorded by the patients (see Section 2.3.4) and the symptoms reported by the patients.

#### 2.3.3. Wearable Sensors

**Figure 1:**
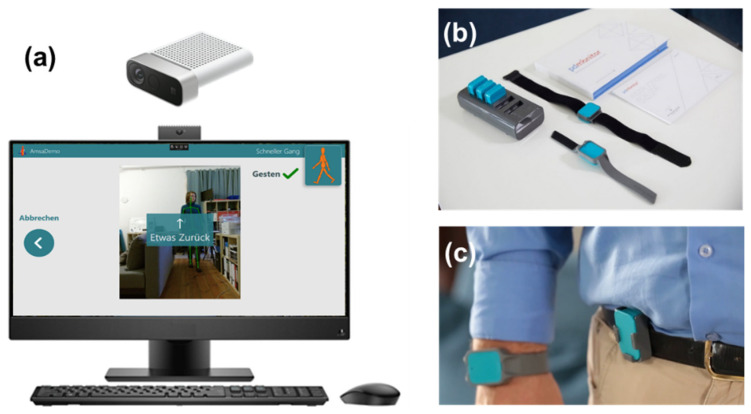
Overview of the sensor system and camera system used in the study. The camera system (**a**) consists of an all-in-one PC with the Motognosis Amsa software installed and a Microsoft Azure Kinect depth camera. The monitor shows prerecorded videos to instruct for the motoric tests. The sensor system (PDMonitor) consists of a smartbox and 5 sensors (**b**), which are attached to each limb and the waist (**c**) and worn for 6 days in a row. Image sources: Motognosis GmbH (**a**) and PD neurotechnology Ltd. (**b**,**c**), used with permission.

During the study, patients used wearable sensors, which are approved as a class 1-m medical device (PDMonitor, PD Neurotechnology Ltd., London, UK, see Figure 1b,c). Five inertial sensors were attached to the patient’s arms, legs, and trunk during the daytime. The sensors recorded and analyzed movement patterns, returning an interpretation of motor status from everyday movements. Patients used the wearable sensors at two time points, each for six days in a row (see Figure 1). Participants agreed that PD Neurotechnology Ltd. uses pseudonymized sensor data and baseline clinical data from the study to improve and develop algorithms.

#### 2.3.4. Camera System

The system consists of a standalone PC with an RGB-depth camera (Microsoft Azure Kinect), with the Motognosis Amsa software installed (Motognosis GmbH, Berlin, Germany, see Figure 1a). Patients are guided by video- and audio-instructions to perform specific motor tasks, which are recorded by the depth camera. Patients performed all the recording sessions independently without personal interaction with the TelePark team. A combination of video-guided MDS-UPDRS III items and the Motognosis Amsa-Protocol (a predefined protocol of the company with different movement tasks) were performed within the scope of the study. Assessments consisted of finger tapping, hand movements, pronation, toe-tapping, leg agility, arising from a chair, gait, freezing of gait, postural stability, posture, postural tremor, kinetic tremor, rest tremor amplitude, and constancy of rest tremor, balance, 360°-turning and stepping in place. Every two weeks, patients were asked to perform this motor assessment, which required approximately 20 min. Kinematic parameters were derived from these tasks to describe patients’ mobility and symptom changes. To ensure the anonymity of the participants, the videos were only accessible to the treating physician. Participants agreed that Motognosis GmbH uses pseudonymized depth data, kinematic parameters, and baseline clinical data to improve and develop algorithms.

### 2.4. Course of the Study

**Figure 2:**
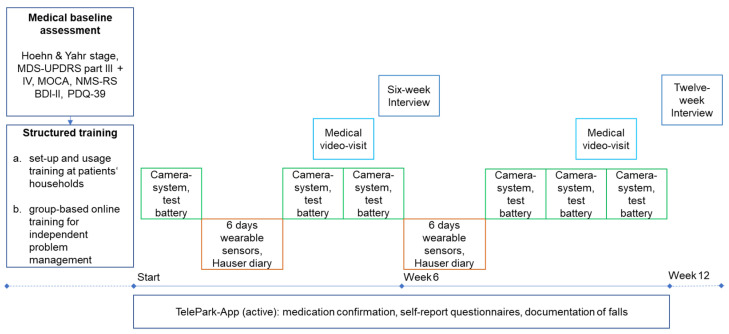
Study design of the TelePark pilot study. The camera system and active tests in the TelePark-App were used in a 2-week interval. The wearable sensors were worn for 6 days in a 6-week interval. The TelePark-App was used continuously to confirm medication intakes and report a daily questionnaire. MDS-UPDRS = Unified Parkinson’s Disease Rating Scale; MOCA = Montreal Cognitive Assessment.

As shown in Figure 2, study participants performed a medical baseline assessment and structured training before the twelve-week study period. The baseline assessment was conducted face-to-face by the treating physician (JB) and included a structured clinical examination and history taking. The following scales and questionnaires were used: Hoehn and Yahr scale [27], Movement Disorder Society Unified Parkinson’s Disease Rating Scale III and IV (MDS-UPDRS) [28], Non-Motor Symptoms Rating Scale (NMS-RS) [29], Montreal Cognitive Assessment (MOCA) [30], PDQ-39, and Beck Depression Inventory-II (BDI-II) [31]. The PDQ-39 was administered to patients a second time at the end of the study.

Before the intervention, patients took part in structured training for each device in their households, followed by a structured online group-based training aimed to allow independent problem-solving. Training videos for each device were created to show possible technical problems and their solutions. Every video was followed by a short quiz to reinforce independent problem-solving. In addition, patients were provided with a booklet that contained step-by-step instructions and troubleshooting.

The intervention included six measurements with the camera-system, two six-day measurements with the wearable sensors, two video consultations, and continuous use of the TelePark-App for confirmation of medication intake and self-report questionnaires. In addition, the TelePark-App was used to run a short active test battery every 2 weeks and to assess motor fluctuations along with each phase of wearing the sensors using a digitized Hauser diary [32]. After six weeks of using all components of the telemedical solution, patients were interviewed with semi-standardized telephone interviews (six-week interview). The aim was to investigate potential technical difficulties and usability issues of the telemedical solution. At the end of the twelve-week intervention, patients’ experience with the telemedical environment and their evaluation of the medical and technical support provided in the study was investigated by an interview with semi-standardized questionnaires.

### 2.5. Evaluation

#### 2.5.1. Semi-Standardized Telephone Interview (Six-Week Interview)

For the six-week interviews with patients, a semi-standardized interview guideline was developed. The questions in these interview guidelines addressed potential barriers to the use of the telemedical solution (technical problems and usability issues) that were based on the results of a comprehensive literature search and previous findings of the TelePark project (e.g., a previous needs analysis). Interviews were recorded using a voice recorder and the interviewees’ responses were documented in an interview protocol. The results were analyzed by the two researchers who conducted the interviews (JM and MSc). Answers to open-ended questions were analyzed by mapping the documented answers and deriving categories inductively. If there were any uncertainties in the data evaluation, they were discussed and resolved by consent within the research team.

#### 2.5.2. Semi-Standardized Telephone Interview (Twelve-Week Interview)

The interview consisted of open-ended and close-ended questions. The closed-ended questions addressed the effort for using the devices, their integration into everyday life, and patients’ satisfaction with different elements of the TelePark intervention. Satisfaction and effort were rated on a five-item Likert-Scale (“Very bad”, “Bad”, “Okay”, “Good”, “Very good”); integration into everyday life with a binary question (yes/no). Open-ended questions asked patients to describe what they liked and did not like about the different elements of the TelePark intervention. The interviewees’ responses were documented by the interviewer in a semi-standardized interview protocol based on the questions of the interview guideline. The results were analyzed by the researcher who conducted the interview (TM), the support-coordinator (AW), and the treating physician (JB) of the TelePark-team. Open-ended questions were analyzed by mapping the answers and deriving categories inductively. Any uncertainties in the data evaluation were discussed and resolved by consent within the research team.

#### 2.5.3. Statistical Analyses

Data are depicted as median with 25th and 75th percentile/range or as mean with standard deviation. The total scores and domain scores of PDQ-39 at the beginning and the end of the study were compared via paired *t*-tests. The adherence of patients to the different components was calculated as the ratio of valid responses to scheduled tasks over the entire study duration (overall adherence) or over one week (weekly adherence). Statistical analyses and data visualization were performed with Python (packages Statsmodels, Scipy, Matplotlib, Seaborn). The sample size of twelve participants was determined using guidelines for the conduction of pilot phase trials [33].

## 3. Results

### 3.1. Demographic Data and Study Completion

Demographic and clinical data are summarized in Table 1. From 12 initially recruited participants, one dropped out immediately after receiving the study devices because of a self-perceived inability to handle the technical requirements. The remaining 11 participants completed the study duration of 12 weeks.

### 3.2. Effects on Quality of Life

The quality of life was measured with the PDQ-39 during the baseline assessment and at the end of the study. We found no significant difference in the total PDQ-39 score between the first and the second measurement (*p* = 0.9972, paired *t*-test)—as expected for a feasibility study with small sample size. Subdomains of the PDQ-39 likewise showed no significant differences (Mobility: *p* = 0.9227, Activities of daily living: *p* = 1.0000, Emotional wellbeing: *p* = 0.9443, Stigma: *p* = 0.7961, Social support: *p* = 0.3607, Cognitions: *p* = 0.4723, Communication: *p* = 0.6018, Bodily discomfort: *p* = 0.8750).

### 3.3. Effects on Medication and Supportive Therapy

**Figure 3:**
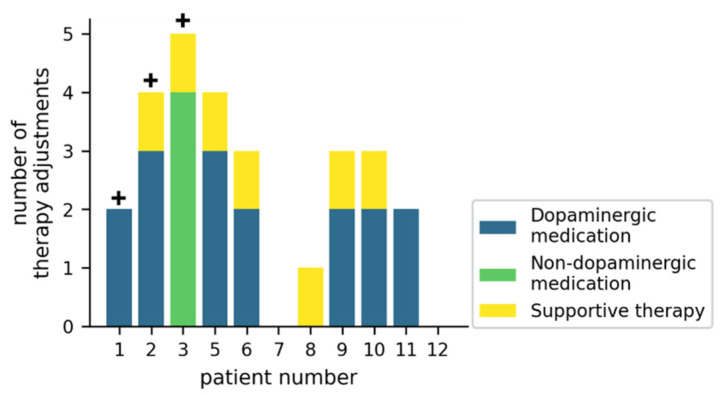
Therapy adjustments during the study for individual patients. The crosses (+) represent patients for which a Multimodal Complex Treatment was scheduled. Supportive therapies include physiotherapy, occupational therapy, or speech-language therapy.

Figure 3 gives an overview of how the therapy was adjusted during the trial. In 7/11 patients, dopaminergic medication was adjusted at least twice, while non-dopaminergic medication was adjusted in 1/11 patients. Supportive therapies (physiotherapy, occupational therapy or speech-language therapy) were initiated or reinitiated in 7/11 patients. For 3/11 patients, an inpatient Multimodal Complex Treatment [34] was scheduled, but did not take place during the study duration.

### 3.4. Contacts with the TelePark-Team

**Figure 4:**
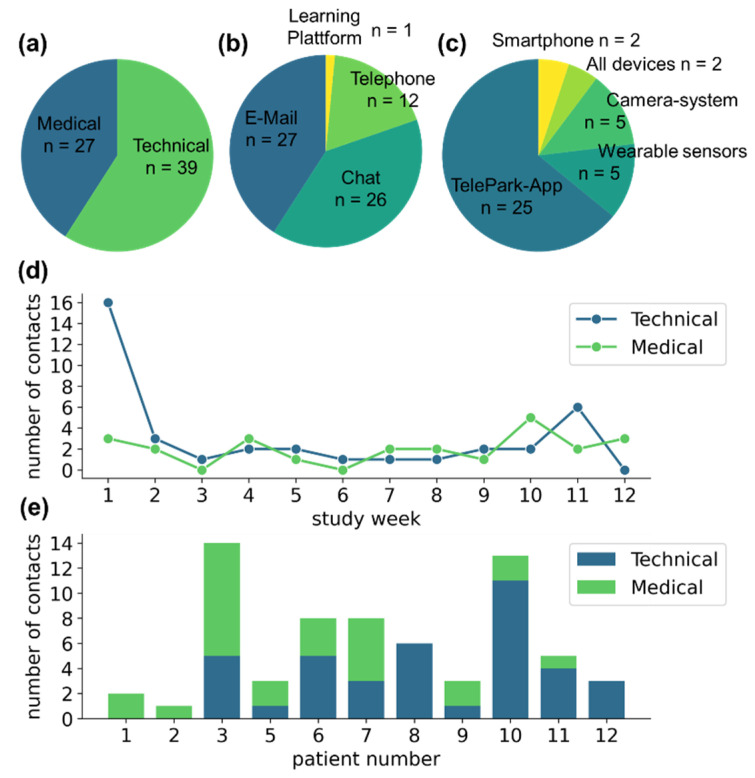
Contact behavior of participants with the TelePark team. (**a**) The absolute number of contacts related to medical and technical issues. (**b**) Means of communication used by the participants. (**c**) Reasons for seeking technical support. (**d**) Contact behavior throughout the study. (**e**) The number of medically or technically motivated contacts for each patient as a stacked barplot.

Participants contacted the TelePark-team 66 times during the study. Of those, 39 of the contacts were related to technical issues and 27 to medical concerns (see Figure 4a). Patients were instructed to preferably use the chat function of the TelePark-App, which was only partially adopted (*n* = 26). Other contacts were performed via email (*n* = 27), and a small fraction via telephone (*n* = 12) or through the learning platform that was used for the structured training sessions (*n* = 1) (see Figure 4b).

The majority of technical issues was associated with the newly developed TelePark-App (*n* = 25), while patients reported only a few technical problems associated with the established camera system and the wearable sensors (*n* = 5 and *n* = 5, respectively) (see Figure 4c). The number of reported technical issues was highest during the first week of the study. Although less frequently, contacts due to technical issues were observed throughout the entire observation period. Medically motivated contacts occurred at a constant rate throughout the study (see Figure 4d). The contact behavior showed high variability between participants (see Figure 4e).

### 3.5. Adherence

**Figure 5:**
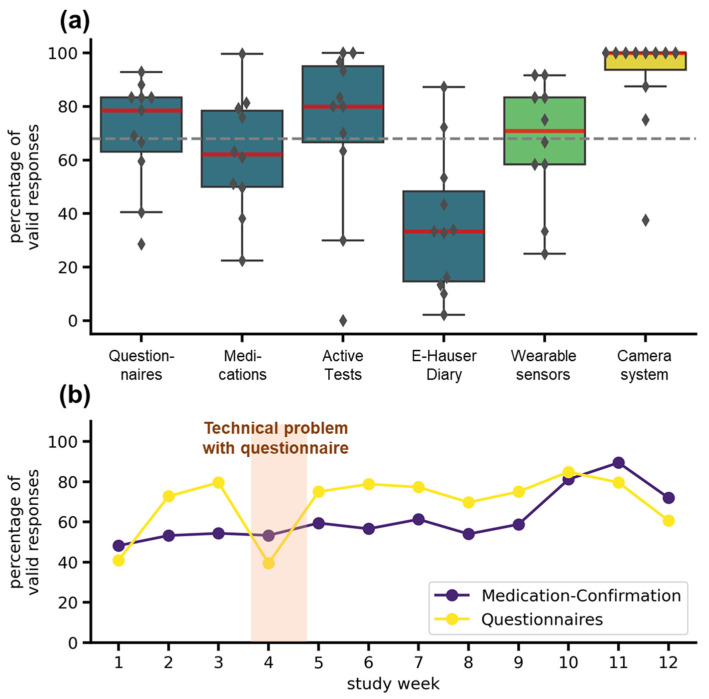
Adherence to different telemedical components. (**a**) Adherence for all telemedical components over the entire study duration. The boxplots show median (red line), interquartile range (boxes), and total range (whiskers). Markers represent adherence for individual patients. The colors indicate the systems: TelePark-App (blue), wearable sensors (light green), and camera-system (yellow). The dashed grey line represents the target completion rate of 68%. (**b**) Weekly adherence to questionnaires and medication confirmations in the TelePark-App throughout the study. The line plot shows the mean percentage of valid confirmations of all patients for each week.

The adherence was highly dependent on the tasks scheduled and on the specific systems used (see Figure 5a). In the TelePark-App, adherence to the short questionnaire every second day was 79%, adherence to active tests and valid medication confirmations was 73% and 62%. The electronic version of the Hauser diary used to rate the motor status every 30 min over 3 days showed the lowest completion rate with 33%. The wearable sensors recorded 71% of valid daily measurements. Missing data was mainly caused by technical problems and not by an unwillingness to wear the devices. Median Adherence for the camera system was 100%, but three patients showed lower adherence (see Figure 5a). Compared to the target completion rate of 68%, derived from a similar trial [22], all except medication confirmation and the electronic Hauser diary met expectations. The adherence to the most frequently occurring assessments (questionnaires and confirmations of medication) did not decrease throughout the study (see Figure 5b).

### 3.6. Participant Experience and Usability

#### 3.6.1. Six-Week Interview

In the six-week interview, patients were asked about technical problems and usability. The least technical problems were reported for the camera-system (4/11) (see Table 2). For the TelePark-App and the wearable sensors, 9/11 and 10/11 participants reported technical problems. For example, they reported that the smartphone’s word recognition system did not work within the app. Additionally, medication reminders or automatic forwardings within the app did not work adequately or were delayed. Concerning the wearable sensors, patients reported data transmission errors or difficulties with inserting the sensors into the smartbox used for charging and data transfer. Additionally, participants missed feedback about successful data transmission.

Difficulties with the legibility or with the recognizability of operating elements occurred in 5/11 patients and difficulties with navigation or fault tolerance occurred in 7/11 patients using the TelePark-App. For example, patients reported that the size of the font was too small and the content of the app was difficult to read. Operating elements were hard to recognize due to insufficient color contrast and difficult to hit because of their small size. For the camera system, difficulties with navigation or fault tolerance were reported once, while difficulties with the legibility of font or with the recognizability of operating elements did not occur. Both aspects were not relevant and investigated for the wearable sensors.

Difficulties in understanding the functioning and operation were more frequently observed with the camera-system (5/11) compared to the TelePark-App (4/11) and the wearable sensors (2/11). For example, patients reported that the tasks described within the camera system sometimes did not fit the presented videos. Furthermore, technical terms were used within the camera system that some patients did not understand. Concerning the TelePark-App, patients reported, for example, misleading user feedback, which resulted in them not knowing what to do next.

#### 3.6.2. Twelve-Week Interview

**Figure 6:**
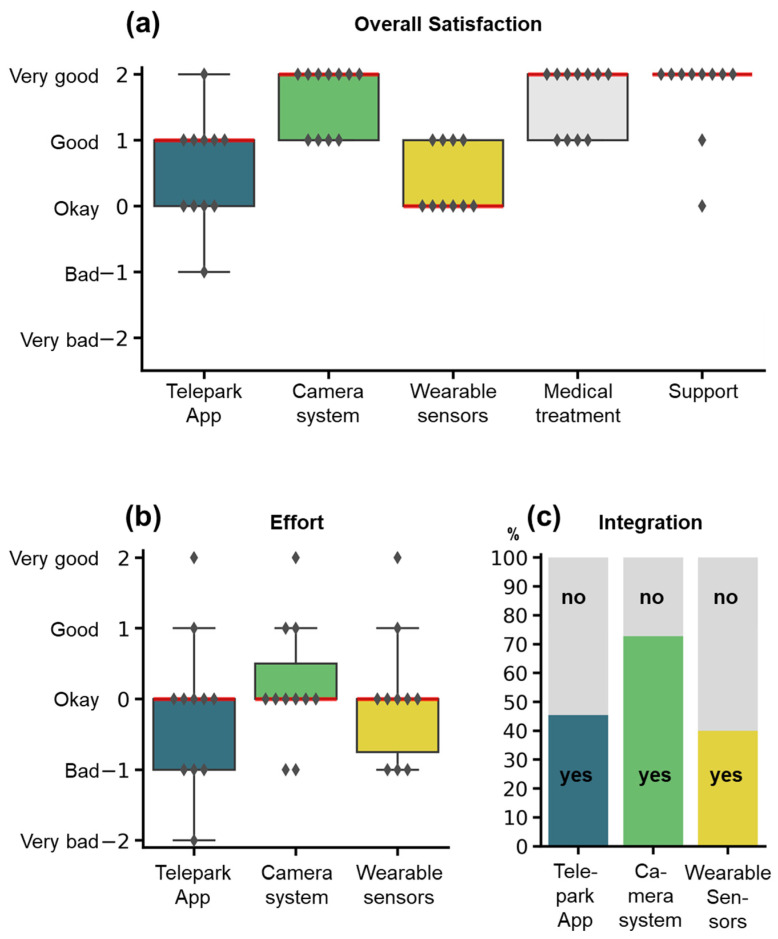
Rating of the study components by the participants. Items were assessed by semi-standardized questionnaires at the end of the study. (**a**) Overall satisfaction for the different study components. (**b**) Effort for using the wearable sensors, the camera-system, or the TelePark-App. (**c**) Integration into the everyday life of the three different systems. Satisfaction and effort were rated on a 5-item Likert scale, integration into everyday life with a binary question (yes/no). Boxplots show median (red line), interquartile range (boxes), and total range (whiskers). Markers represent responses for individual patients. The stacked barplot shows percentages of the answers yes (colors) or no (grey).

In the twelve-week interview, participants were asked to rate each system as well as medical treatment and technical support on a five-item Likert-scale with the following question: “All in all, how did you like...” (Figure 6a). The camera-system, the wearable sensors, and the TelePark-App were additionally scored for effort and integration into everyday life (yes/no). The overall satisfaction was rated “Okay” to “Very good” for the different components. The amount of effort for using the individual systems (Figure 6b) was justifiable from the patient perspective (Median: “Okay” for all systems). However, many patients reported, that the combination of all three systems resulted in high efforts for complying with all study procedures. Only a fraction of patients felt that the systems were integrated well into their everyday lives (Figure 6c).

The twelve-week interview further included open-ended questions about positive and negative aspects of each telemedical component. The following categories could be derived from our analysis: perspicuity, flexibility, novelty, dependability, burden, wearing comfort, technical problems, transparency, fast communication and easy accessibility, space requirement, expected diagnostic value, stimulation, efficacy, educational aspects, and stigma. Most frequently, participants mentioned as positive aspects perspicuity (*n* = 14), novelty (*n* = 8), and the possibility to contact the TelePark-team fast and easily (*n* = 3). The negative aspects were lack of flexibility (*n* = 9), technical problems (*n* = 7), low wearing comfort (*n* = 6), low dependability (*n* = 5), high usage burden (*n* = 5), problems with perspicuity (*n* = 4), and a lack of transparency (*n* = 4).

Despite their advanced age (65 y, SD = 9.2), patients emphasized the novelty of the systems and were interested in experimenting with previously unknown concepts like gesture control:

Pat_02: *“I found the exercises [from the camera-system] and also the gesture control very interesting.”*

The low wearing comfort was specifically associated with the wearable sensors, for which patients frequently reported that the straps for attaching the sensors were not comfortable enough:

Pat_06: *“Now that I’m not exactly slim built, the belly sensor was a little too tight for me.”*

One patient even associated the body-worn sensors with stigmatization in public:

Pat_09: *“I often received silly looks from outsiders.”*

For the TelePark-App, a frequent negative aspect was technical problems that caused the majority of support contacts with the TelePark team. As this was a newly developed system, the main problems of the TelePark-App were associated with a lack of flexibility, technical problems, low perspicuity, and a high usage burden. Medication confirmation and digital Hauser-diary were generally mentioned as very demanding and irritating, which resulted in low adherence rates:

Pat_03: *“The constant beeping [of the smartphone], no matter where you were, that was of course annoying over time.”*

The camera system had a good adherence rate and was associated with low burden, high novelty, and good perspicuity. For the camera system, the bulkiness was a recurring system-specific drawback.

## 4. Discussion

The main findings of our study were: (i) The TelePark intervention is feasible in our cohort of mainly elderly patients with PD. (ii) Patient adherence was acceptable for most assessments and stable throughout the pilot study. (iii) Technical support is necessary over the entire study duration. (iv) Specific limits for telemedical systems (space requirements, wearing comfort, burden) can be identified best by testing in a real-life environment.

This study is a pilot trial for a subsequent randomized trial that will test the clinical benefit of a sensor-assisted telemedicine intervention. The aims of the pilot trial were to determine (1) technical feasibility, (2) usability by patients, and (3) potential for therapeutic efficacy. Concerning technical feasibility, one relevant problem occurred in week 4, which was resolved by minor modifications in the software. Despite extensive training at the start of the study, the majority of contact requests were motivated by technical, and not medical concerns (59% and 41%, respectively). The steep decline of technical requests after the first week of the study shows that patients adapted rapidly to using a variety of novel technical systems in their home environment. This observation is consistent with results by others showing high motivation to engage with new technology despite an initially low technology literacy among elderly persons [35]. Nevertheless, our results indicate that technical support is needed throughout the entire course of telemedical interventions since technical problems were encountered every week of the study. The low percentage of chat usage by patients (39%) could be explained by a combination of low familiarity and lack of necessity. In this regard, results from a similar study are comparable to our findings [36].

With respect to usability by patients, we observed a high completion rate of digital sensor tasks, especially for the less burdensome camera-system (100%). Completion rates were lower for more laborious tasks such as the digital Hauser-diary (33%). Overall adherence rates were comparable to similar studies [22,36]. We saw stable adherence to medication confirmations throughout the study (overall adherence 62%), but all patients reported that medication confirmations in the TelePark-App did not reflect their true medication intake. Although medication alerts and digital confirmations can potentially increase adherence to medication schedules [37], this discrepancy should caution researchers to rely on digital medication-adherence in clinical practice and clinical trials.

One patient (out of 12) dropped out of the study, which is comparable or better than similar interventions [19,22,36,38], consistent with the generally high satisfaction rating at the end of the study. The fact that the patient dropped out just before he had to start using the devices could indicate that the requirements were only fully understood after the training sessions were finished. This suggests that telemedicine and digital technologies are at present not suited for all patients. We hypothesized that attrition might compromise the utility of digital monitoring. Yet, no patient dropped out throughout the active phase of the study, and completion rates remained stable. We hypothesize that the personal connection to a dedicated support coordinator and close contact with the treating physician were central aspects of the high completion rates.

The interviews at the end of the study suggest that the concepts of perspicuity, good wearing comfort, low usage-burden, flexibility, and transparency are central to patient satisfaction and long-term adherence. The domains of flexibility and transparency indicate that patients want to better understand their symptoms and adapt the technology used to monitor symptoms to their personal needs. This highlights the importance of patient-centeredness when developing devices or applications for patients. The remaining concepts can be synthesized under a general ease-of-use domain, complying with guidelines [39,40], but emphasizing usability from a patient perspective.

Based on the findings of this pilot trial, the tasks “medication confirmation” and “digital Houser diary” were removed from the TelePark-App for the subsequent randomized trial because they were considered effortful, showed adherence rates under the cutoff score, and were not absolutely necessary. For the same reason, several additional questionnaires were dropped. The font size and clarity of the TelePark-App were improved. To further reduce patients’ effort, the subsequent randomized trial will combine the TelePark-App with only one of camera-system or wearable sensors, and not both.

In the small cohort of this pilot trial, the quality of life (PDQ-39 scores) did not change significantly. This can be explained by the small sample size and the short study duration of only three months. Furthermore, all patients recruited into this pilot trial were already treated by a movement disorder specialist regularly, potentially limiting the range of improvement.

The strengths of our study were the variety of systems tested together in a real-life environment and the use of closed-ended and open-ended questions within semi-standardized personal telephone interviews after six and twelve weeks of use. This allowed a comparison between component-specific adherence and the identification of contributing factors. Moreover, the tracking of contacts with the TelePark-team allowed us to draw conclusions about the required professional staff in telemedical interventions. The limitations of our study were the small sample size and the duration of three months, which could result in underrepresentation of certain patient groups or missed long-term effects. The inclusion of only one patient with mild cognitive impairment (MOCA = 24) in the study cohort further limits the generalizability of our results. The patient with MCI showed similar adherence rates compared with other patients in all tasks except the active tests in the TelePark-App.

## 5. Conclusions

Overall, conducting a trial with a multimodal telemedical intervention that combines video visits, a smartphone app, a camera system, and wearable sensors is feasible. The one drop-out suggests that patients need to be selected and informed well for such interventions. The number of contacts for technical questions highlights the requirement to provide technical support during the intervention in addition to the medical support. Assessments and questionnaires need to be reduced to a minimum to maintain motivation for the trial. A telemedicine intervention can allow earlier medication changes, but not better motor outcomes by itself, which is important for choosing trial duration and outcomes. Sensor-based assessments can report symptoms in more detail and potentially allow more efficacious treatment recommendations. Health-related quality of life is probably the most valid outcome for such trials. Patient-centered usability and patient empowerment need to guide the development of digital health solutions.

## Figures and Tables

**Table 1:** Demographic data of the study cohort.

Variable	Study Cohort
Number of patients, *n*	11
Age, mean (SD)	65 (9.2)
Sex, *n*: female; male	5; 6
Hoehn and Yahr stage, median (Range)	2 (1–4)
Disease duration, mean (SD)	7 (5.7)
MDS-UPDRS III score, mean (SD)	30 (18.1)
MDS-UPDRS IV score, mean (SD)	4 (6.0)
NMS-RS score, mean (SD)	70 (34.3)
MOCA score, mean (SD)	27 (2.9)
BDI-II score, mean (SD)	15 (10.7)
PDQ-39 score, mean (SD)	20 (9.5)

**Table 2:** Results of the six-weeks interview (*n* = 11).

	TelePark-App	Wearable Sensors	Camera-System
Frequency of difficulties * concerning			
Technical operability, *n*	10	9	4
Legibility of the font/recognizability of operating elements, *n*	5	NA	0
Navigation/fault tolerance, *n*	7	NA	1
Understanding the functioning, *n*	4	2	5

* Multiple responses possible.

## Data Availability

Data generated or analyzed in the study are available from the authors upon reasonable request.

## References

[1] Ray Dorsey E., Elbaz A., Nichols E., Abd-Allah F., Abdelalim A., Adsuar J.C., Ansha M.G., Brayne C., Choi J.Y.J., Collado-Mateo D. (2018). Global, Regional, and National Burden of Parkinson’s Disease, 1990–2016: A Systematic Analysis for the Global Burden of Disease Study 2016. Lancet. Neurol..

[2] Rossi A., Berger K., Chen H., Leslie D., Mailman R.B., Huang X. (2018). Projection of the Prevalence of Parkinson’s Disease in the Coming Decades: Revisited. Mov. Disord..

[3] Wanneveich M., Moisan F., Jacqmin-Gadda H., Elbaz A., Joly P. (2018). Projections of Prevalence, Lifetime Risk, and Life Expectancy of Parkinson’s Disease (2010–2030) in France. Mov. Disord..

[4] Singh R.L., Bush E.J., Mary Jo Cooley H., Phillips Carrico C., Sundin S. (2020). Considering Health Care Needs in a Rural Parkinson Disease Community. Prog. Community Health Partnersh. Res. Educ. Action.

[5] Dorsey E.R., Bloem B.R. (2018). The Parkinson Pandemic—A Call to Action. JAMA Neurol..

[6] Powers R., Etezadi-Amoli M., Arnold E.M., Kianian S., Mance I., Gibiansky M., Trietsch D., Alvarado A.S., Kretlow J.D., Herrington T.M. (2021). Smartwatch Inertial Sensors Continuously Monitor Real-World Motor Fluctuations in Parkinson’s Disease. Sci. Transl. Med..

[7] Del Din S., Galna B., Godfrey A., Bekkers E.M.J., Pelosin E., Nieuwhof F., Mirelman A., Hausdorff J.M., Rochester L. (2019). Analysis of Free-Living Gait in Older Adults With and Without Parkinson’s Disease and With and Without a History of Falls: Identifying Generic and Disease-Specific Characteristics. J. Gerontol. Ser. A.

[8] Ossig C., Gandor F., Fauser M., Bosredon C., Churilov L., Reichmann H., Horne M.K., Ebersbach G., Storch A. (2016). Correlation of Quantitative Motor State Assessment Using a Kinetograph and Patient Diaries in Advanced PD: Data from an Observational Study. PLoS ONE.

[9] Omberg L., Chaibub Neto E., Perumal T.M., Pratap A., Tediarjo A., Adams J., Bloem B.R., Bot B.M., Elson M., Goldman S.M. (2021). Remote Smartphone Monitoring of Parkinson’s Disease and Individual Response to Therapy. Nat. Biotechnol..

[10] Lipsmeier F., Taylor K.I., Kilchenmann T., Wolf D., Scotland A., Schjodt-Eriksen J., Cheng W.Y., Fernandez-Garcia I., Siebourg-Polster J., Jin L. (2018). Evaluation of Smartphone-Based Testing to Generate Exploratory Outcome Measures in a Phase 1 Parkinson’s Disease Clinical Trial. Mov. Disord..

[11] Myers T.L., Tarolli C.G., Adams J.L., Barbano R., Cristina Gil-Díaz M., Spear K.L., Lowell J., Daeschler M., Riley L., Amondikar N. (2021). Video-Based Parkinson’s Disease Assessments in a Nationwide Cohort of Fox Insight Participants. Clin. Park. Relat. Disord..

[12] Beck C.A., Beran D.B., Biglan K.M., Boyd C.M., Dorsey E.R., Schmidt P.N., Simone R., Willis A.W., Galifianakis N.B., Katz M. (2017). National Randomized Controlled Trial of Virtual House Calls for Parkinson Disease. Neurology.

[13] Khan T., Zeeshan A., Dougherty M. (2021). A Novel Method for Automatic Classification of Parkinson Gait Severity Using Front-View Video Analysis. Technol. HealthCare.

[14] Liu Y., Chen J., Hu C., Ma Y., Ge D., Miao S., Xue Y., Li L. (2019). Vision-Based Method for Automatic Quantification of Parkinsonian Bradykinesia. IEEE Trans. Neural. Syst. Rehabil. Eng..

[15] Jin B., Qu Y., Zhang L., Gao Z. (2020). Diagnosing Parkinson Disease Through Facial Expression Recognition: Video Analysis. J. Med. Internet Res..

[16] Sica M., Tedesco S., Crowe C., Kenny L., Moore K., Timmons S., Barton J., O’Flynn B., Komaris D.S. (2021). Continuous Home Monitoring of Parkinson’s Disease Using Inertial Sensors: A Systematic Review. PLoS ONE.

[17] Sibley K.G., Girges C., Hoque E., Foltynie T. (2021). Video-Based Analyses of Parkinson’s Disease Severity: A Brief Review. J. Park. Dis..

[18] Little M.A. (2021). Smartphones for Remote Symptom Monitoring of Parkinson’s Disease. J. Park. Dis..

[19] Gatsios D., Antonini A., Gentile G., Marcante A., Pellicano C., MacChiusi L., Assogna F., Spalletta G., Gage H., Touray M. (2020). Feasibility and Utility of MHealth for the Remote Monitoring of Parkinson Disease: Ancillary Study of the PD_manager Randomized Controlled Trial. JMIR mHealth uHealth.

[20] Isaacson S.H., Boroojerdi B., Waln O., McGraw M., Kreitzman D.L., Klos K., Revilla F.J., Heldman D., Phillips M., Terricabras D. (2019). Effect of Using a Wearable Device on Clinical Decision-Making and Motor Symptoms in Patients with Parkinson’s Disease Starting Transdermal Rotigotine Patch: A Pilot Study. Park. Relat. Disord..

[21] Van den Bergh R., Bloem B.R., Meinders M.J., Evers L.J.W. (2021). The State of Telemedicine for Persons with Parkinson’s Disease. Curr. Opin. Neurol..

[22] De Lima A.L.S., Hahn T., Evers L.J.W., De Vries N.M., Cohen E., Afek M., Bataille L., Daeschler M., Claes K., Boroojerdi B. (2017). Feasibility of Large-Scale Deployment of Multiple Wearable Sensors in Parkinson’s Disease. PLoS ONE.

[23] Jenkinson C., Fitzpatrick R., Peto V., Greenhall R., Hyman N. (1997). The Parkinson’s Disease Questionnaire (PDQ-39): Development and Validation of a Parkinson’s Disease Summary Index Score. Age Ageing.

[24] Berger K., Broll S., Winkelmann J., Heberlein I., Müller T., Ries V. (1999). Reliability Analysis of the PDQ-39 (German Version). Aktuelle Neurol..

[25] Postuma R.B., Berg D., Stern M., Poewe W., Olanow C.W., Oertel W., Obeso J., Marek K., Litvan I., Lang A.E. (2015). MDS Clinical Diagnostic Criteria for Parkinson’s Disease. Mov. Disord..

[26] Dalrymple-Alford J.C., MacAskill M.R., Nakas C.T., Livingston L., Graham C., Crucian G.P., Melzer T.R., Kirwan J., Keenan R., Wells S. (2010). The MoCA. Neurology.

[27] Hoehn M.M., Yahr M.D. (1967). Parkinsonism: Onset, Progression and Mortality. Neurology.

[28] Goetz C.G., Tilley B.C., Shaftman S.R., Stebbins G.T., Fahn S., Martinez-Martin P., Poewe W., Sampaio C., Stern M.B., Dodel R. (2008). Movement Disorder Society-Sponsored Revision of the Unified Parkinson’s Disease Rating Scale (MDS-UPDRS): Scale Presentation and Clinimetric Testing Results. Mov. Disord..

[29] Martinez-Martin P., Rodriguez-Blazquez C., Kurtis M.M., Chaudhuri K.R. (2011). The Impact of Non-Motor Symptoms on Health-Related Quality of Life of Patients with Parkinson’s Disease. Mov. Disord..

[30] Nasreddine Z.S., Phillips N.A., Bédirian V., Charbonneau S., Whitehead V., Collin I., Cummings J.L., Chertkow H. (2005). The Montreal Cognitive Assessment, MoCA: A Brief Screening Tool for Mild Cognitive Impairment. J. Am. Geriatr. Soc..

[31] Beck A.T., Steer R.A., Ball R., Ranieri W.F. (1996). Comparison of Beck Depression Inventories -IA and -II in Psychiatric Outpatients. J. Pers. Assess..

[32] Hauser R.A., Friedlander J., Zesiewicz T.A., Adler C.H., Seeberger L.C., O’Brien C.F., Molho E.S., Factor S.A. (2000). A Home Diary to Assess Functional Status in Patients with Parkinson’s Disease with Motor Fluctuations and Dyskinesia. Clin. Neuropharmacol..

[33] Julious S.A. (2005). Sample Size of 12 per Group Rule of Thumb for a Pilot Study. Pharm. Stat..

[34] Hartelt E., Scherbaum R., Kinkel M., Gold R., Muhlack S., Tönges L. (2020). Parkinson’s Disease Multimodal Complex Treatment (PD-MCT): Analysis of Therapeutic Effects and Predictors for Improvement. J. Clin. Med..

[35] Wang S., Bolling K., Mao W., Reichstadt J., Jeste D., Kim H.-C., Nebeker C. (2019). Technology to Support Aging in Place: Older Adults’ Perspectives. Healthcare.

[36] Bouça-machado R., Pona-ferreira F., Leitão M., Clemente A., Vila-viçosa D., Kauppila L.A., Costa R.M., Matias R., Ferreira J.J. (2021). Feasibility of a Mobile-based System for Unsupervised Monitoring in Parkinson’s Disease. Sensors.

[37] Pérez-Jover V., Sala-González M., Guilabert M., Mira J.J. (2019). Mobile Apps for Increasing Treatment Adherence: Systematic Review. J. Med. Internet Res..

[38] Lakshminarayana R., Wang D., Burn D., Chaudhuri K.R., Galtrey C., Guzman N.V., Hellman B., James B., Pal S., Stamford J. (2017). Using a Smartphone-Based Self-Management Platform to Support Medication Adherence and Clinical Consultation in Parkinson’s Disease. NPJ Park. Dis..

[39] Llorens-Vernet P., Miró J. (2020). Standards for Mobile Health-Related Apps: Systematic Review and Development of a Guide. JMIR mHealth uHealth.

[40] Chatzipavlou I.A., Christoforidou S.A., Vlachopoulou M. (2016). A Recommended Guideline for the Development of MHealth Apps. mHealth.

